# Supplementary material for: Severity Detection for the Coronavirus Disease 2019 (COVID-19) Patients Using a Machine Learning Model Based on the Blood and Urine Tests
Source: Front Cell Dev Biol. 2020 Jul 31;8:683. doi: 10.3389/fcell.2020.00683 (PMC7411005; doi:10.3389/fcell.2020.00683)
Supplement: Supplementary file 1 [file Table_1.DOCX]

# Supplementary Table S1

# Detailed information of all the 100 features collected for the COVID-19 infected patients. The columns “MeanP” and “StdP” were the mean and standard deviation of this variable in the positive samples. The columns “MeanN” and “StdN” were these two metrics of the negative samples. The column “Pvalue” gave the T-test Pvalue of how significantly each feature discriminate the positive samples from the negative ones

| **Feature** | **Pvalue** | **MeanP** | **StdP** | **MeanN** | **StdN** |
| --- | --- | --- | --- | --- | --- |
| Age | 1.75E-06 | 65.0533 | 14.5462 | 51.4839 | 17.2196 |
| Diastolic pressure | 6.24E-01 | 61.0400 | 33.5223 | 63.8226 | 32.3006 |
| Heart rate(HR) | 9.81E-01 | 80.6133 | 46.0865 | 80.4516 | 29.5744 |
| Respiratory rate(RR) | 3.04E-01 | 21.3867 | 12.6137 | 19.4839 | 7.9046 |
| Sex | 7.71E-05 | 0.2133 | 0.4124 | 0.5323 | 0.5030 |
| Systolic pressure | 6.33E-01 | 105.8667 | 55.2235 | 101.4516 | 51.7689 |
| Temperature(T) | 1.67E-01 | 33.5320 | 11.7045 | 35.8548 | 6.6469 |
| Blood \| Activated partial thromboplastin time | 1.53E-01 | 30.6527 | 28.2687 | 39.0579 | 40.0452 |
| Blood \| Actual bicarbonate | 1.07E-01 | 23.4120 | 3.1922 | 24.1274 | 1.5067 |
| Blood \| Alanine aminotransferase | 2.74E-02 | 38.8013 | 26.8843 | 29.7710 | 18.8541 |
| Blood \| Albumin | 4.01E-01 | 65.7480 | 112.5770 | 52.5597 | 55.2238 |
| Blood \| Alkaline phosphatase | 8.17E-03 | 61.7413 | 41.5189 | 46.9145 | 14.1322 |
| Blood \| Aspartase aminotransferase | 3.57E-02 | 55.9067 | 91.3692 | 30.5097 | 25.1476 |
| Blood \| Basophil Number | 4.71E-01 | 0.0148 | 0.0196 | 0.0126 | 0.0156 |
| Blood \| Basophil percentage | 6.14E-04 | 2.4707 | 3.3638 | 4.8984 | 4.7174 |
| Blood \| Blood urea nitrogen | 4.83E-01 | 51.2751 | 98.7156 | 64.2194 | 116.5630 |
| Blood \| Calcium | 9.55E-10 | 2.0665 | 0.1393 | 2.2119 | 0.1149 |
| Blood \| Chlorine | 2.25E-02 | 103.6867 | 9.4446 | 100.6016 | 5.1009 |
| Blood \| Cholesterol | 8.87E-01 | 36.6113 | 53.0346 | 35.4524 | 39.0180 |
| Blood \| Complement C3 | 5.83E-02 | 0.9223 | 0.1885 | 0.9758 | 0.1261 |
| Blood \| Complement C4 | 9.75E-01 | 0.2724 | 0.0749 | 0.2727 | 0.0460 |
| Blood \| Direct bilirubin | 5.69E-01 | 52.3573 | 66.6443 | 46.8882 | 38.7827 |
| Blood \| Eosinophil Number | 1.57E-01 | 0.0788 | 0.1296 | 0.1252 | 0.2436 |
| Blood \| Eosinophil percentage | 3.92E-03 | 0.4564 | 0.6717 | 0.7840 | 0.6235 |
| Blood \| Erythrocyte sedimentation rate | 8.54E-02 | 32.0000 | 25.8870 | 24.2742 | 26.0788 |
| Blood \| Fibrinogen | 5.79E-02 | 10.8361 | 6.7575 | 12.8763 | 5.4848 |
| Blood \| Globulin | 6.40E-01 | 23.1167 | 16.3459 | 21.8097 | 16.1543 |
| Blood \| Glucose | 1.92E-04 | 9.1617 | 4.8742 | 6.4194 | 3.0993 |
| Blood \| Haemoglobin | 9.20E-02 | 126.1133 | 21.8770 | 132.2823 | 20.3050 |
| Blood \| High sensitive cardiac troponin I | 5.42E-02 | 525.7153 | 2005.3045 | 30.5274 | 63.2690 |
| Blood \| hs-CRP | 8.01E-05 | 79.5920 | 93.2775 | 28.2694 | 37.4027 |
| Blood \| Immunoglobulin A | 6.61E-01 | 2.5100 | 0.8282 | 2.4549 | 0.5917 |
| Blood \| Immunoglobulin G | 4.50E-01 | 11.4702 | 2.4971 | 12.2477 | 8.4590 |
| Blood \| Immunoglobulin M | 5.45E-01 | 1.6287 | 1.4343 | 1.5132 | 0.4769 |
| Blood \| Indirect bilirubin | 1.20E-01 | 134.7013 | 171.0896 | 179.2903 | 159.6188 |
| Blood \| Interleukin-10 | 1.75E-03 | 12.1500 | 9.4170 | 8.1435 | 3.2677 |
| Blood \| Interleukin-1Œ≤ | 3.75E-01 | 17.3213 | 109.7595 | 4.8919 | 3.1836 |
| Blood \| Interleukin-2R | 7.87E-04 | 814.0819 | 568.0376 | 548.4194 | 240.4630 |
| Blood \| Interleukin-6 | 1.55E-01 | 120.2820 | 577.6147 | 15.1644 | 17.6388 |
| Blood \| Interleukin-8 | 3.97E-01 | 40.9200 | 50.3384 | 33.8887 | 45.6106 |
| Blood \| International normalized ratio | 1.45E-01 | 2.6497 | 1.8348 | 3.1121 | 1.8359 |
| Blood \| Leucocytes | 2.03E-04 | 10.5607 | 11.0356 | 5.1461 | 1.7960 |
| Blood \| Lymphocyte Number | 6.43E-01 | 1.3355 | 6.0575 | 0.9740 | 1.0137 |
| Blood \| Lymphocyte percentage | 4.36E-01 | 9.0184 | 8.5835 | 10.3365 | 11.1368 |
| Blood \| MB isoenzyme of creatine kinase | 3.02E-01 | 8.4760 | 12.9121 | 6.7661 | 1.5466 |
| Blood \| Mean corpuscular hemoglobin | 3.67E-01 | 30.5235 | 2.7717 | 30.9226 | 2.3033 |
| Blood \| Mean corpuscular hemoglobin concentration | 5.56E-01 | 340.1333 | 14.6198 | 337.0968 | 41.5348 |
| Blood \| Mean corpuscular volume | 8.29E-01 | 87.8196 | 12.5105 | 88.2081 | 7.1990 |
| Blood \| Mean platelet volume | 6.55E-02 | 11.0867 | 0.9194 | 10.7823 | 0.9970 |
| Blood \| Monocyte Number | 3.47E-01 | 0.7009 | 3.9029 | 0.2319 | 0.2552 |
| Blood \| Monocyte percentage | 1.62E-05 | 8.6567 | 9.7006 | 17.3052 | 12.9063 |
| Blood \| Myoglobin | 1.36E-01 | 223.7693 | 270.1929 | 167.1274 | 135.9576 |
| Blood \| Neutrophil Number | 1.12E-04 | 4.9155 | 5.6546 | 1.8997 | 2.0824 |
| Blood \| Neutrophil percentage | 4.14E-11 | 82.5440 | 14.5598 | 58.7823 | 23.7638 |
| Blood \| Œ≥-glutamyl transpeptidase | 1.79E-01 | 65.4560 | 64.3722 | 53.7500 | 24.8846 |
| Blood \| Oxygen partial pressure | 7.99E-03 | 86.9493 | 20.6097 | 96.6613 | 21.4917 |
| Blood \| Oxygen saturation | 2.28E-01 | 74.0179 | 39.8537 | 81.7367 | 33.5497 |
| Blood \| Packed cell volume | 8.90E-04 | 2.1565 | 8.8044 | 10.4549 | 18.8088 |
| Blood \| Partial pressure of carbon dioxide | 5.96E-02 | 38.2947 | 4.9081 | 39.5371 | 1.7011 |
| Blood \| PH(blood) | 9.30E-01 | 7.3988 | 0.0560 | 7.3982 | 0.0171 |
| Blood \| Phosphorus | 8.92E-01 | 1.0819 | 0.1884 | 1.0858 | 0.1411 |
| Blood \| Platelet | 7.31E-02 | 175.3067 | 73.7601 | 195.9839 | 56.9754 |
| Blood \| Platelet distribution width | 4.56E-03 | 13.3880 | 2.3615 | 11.7703 | 4.1052 |
| Blood \| Potassium | 2.28E-01 | 4.3659 | 0.8477 | 4.2140 | 0.5570 |
| Blood \| Prothrombin activity | 2.69E-01 | 21.0296 | 22.7598 | 25.0339 | 18.6504 |
| Blood \| Prothrombin time | 2.08E-03 | 9.3161 | 7.8345 | 5.4910 | 6.0838 |
| Blood \| RBC distribution width CV | 4.27E-02 | 13.2747 | 1.9499 | 12.6839 | 1.2840 |
| Blood \| RBC distribution width SD | 1.59E-03 | 42.7053 | 4.8451 | 40.0484 | 4.7498 |
| Blood \| Red blood cell | 1.09E-01 | 4.2148 | 0.6349 | 4.3882 | 0.6172 |
| Blood \| Residual alkali in extracellular fluid | 1.58E-01 | 1.3600 | 4.2525 | 2.2177 | 2.3298 |
| Blood \| Serum creatinine | 9.21E-01 | 66.3253 | 108.7883 | 63.9645 | 168.4702 |
| Blood \| Serum ferritin | 3.62E-03 | 1103.2307 | 1428.1607 | 534.0065 | 548.3254 |
| Blood \| Serum levels of procalcitonin | 8.45E-02 | 0.7779 | 3.2945 | 0.0503 | 0.0394 |
| Blood \| Sodium | 2.90E-02 | 141.4040 | 8.7149 | 138.6210 | 5.2291 |
| Blood \| Standard bicarbonate | 2.57E-01 | 22.5773 | 2.3285 | 22.9419 | 1.0658 |
| Blood \| Thrombin time | 2.78E-05 | 16.4720 | 3.3191 | 14.4548 | 1.6938 |
| Blood \| Total bilirubin | 7.39E-04 | 12.8585 | 14.8919 | 6.1739 | 3.5119 |
| Blood \| Total carbon dioxide | 8.38E-03 | 26.2840 | 5.4694 | 28.8984 | 5.9523 |
| Blood \| Total protein | 1.20E-01 | 51.4360 | 17.0859 | 46.4419 | 20.2562 |
| Blood \| Tube counting | 5.19E-01 | 0.9613 | 0.3175 | 0.9210 | 0.4122 |
| Blood \| Tumor necrosis factor-Œ± | 1.71E-02 | 11.0507 | 8.3826 | 8.3726 | 2.6694 |
| Blood \| Uric acid | 3.05E-01 | 147.6573 | 203.9810 | 115.3694 | 153.1798 |
| Blood \| Whole blood surplus alkali | 1.52E-01 | 1.3533 | 4.3457 | 2.2355 | 2.2847 |
| Urine \| Crystal | 5.03E-01 | 0.0267 | 0.1622 | 0.0484 | 0.2163 |
| Urine \| Ketone bodies | 8.97E-02 | 0.1933 | 0.5192 | 0.0726 | 0.2189 |
| Urine \| Morphology of erythrocytes | 1.53E-01 | 0.0933 | 0.2929 | 0.0323 | 0.1781 |
| Urine \| Pathological tube type | 3.21E-01 | 0.2213 | 0.0894 | 0.2419 | 0.1499 |
| Urine \| PH(Urine) | 4.25E-02 | 6.1633 | 0.3275 | 6.2661 | 0.2432 |
| Urine \| Proportion | 4.67E-01 | 1.0194 | 0.0049 | 1.0186 | 0.0080 |
| Urine \| Red blood cell(occult) | 2.98E-02 | 0.4400 | 0.8052 | 0.1774 | 0.5364 |
| Urine \| Small round epithelial cell | 1.19E-01 | 0.0000 | 0.0000 | 0.0323 | 0.1781 |
| Urine \| Total epithelial cells | 9.11E-01 | 16.4507 | 7.4572 | 16.2903 | 9.2630 |
| Urine \| Urinary bilirubin | 3.65E-01 | 0.0133 | 0.1155 | 0.0000 | 0.0000 |
| Urine \| Urinary gallbladder | 4.91E-01 | 0.2960 | 0.8995 | 0.4129 | 1.0815 |
| Urine \| Urine glucose | 2.78E-01 | 0.2800 | 0.8106 | 0.1452 | 0.5963 |
| Urine \| Urine leukocyte count | 5.75E-01 | 20.7880 | 23.1057 | 26.7129 | 87.7615 |
| Urine \| Urine nitrite | 3.65E-01 | 0.0133 | 0.1155 | 0.0000 | 0.0000 |
| Urine \| Urine protein | 8.41E-03 | 0.4467 | 0.7692 | 0.1613 | 0.3708 |
| Urine \| Urine RBC count | 7.82E-01 | 26.6773 | 52.0145 | 31.7339 | 147.0336 |
| Urine \| White blood cell | 8.28E-01 | 0.1133 | 0.3637 | 0.1290 | 0.4785 |
